# Cross - cultural adaptation and preliminary validation of the Turkish version of the Early Childhood Oral Health Impact Scale among 5-6-year-old children

**DOI:** 10.1186/1477-7525-9-118

**Published:** 2011-12-22

**Authors:** Kadriye Peker, Ömer Uysal, Gülçin Bermek

**Affiliations:** 1 Department of Dental Public Health, Faculty of Dentistry, Istanbul University, 34093 Fatih/Çapa - Istanbul, Turkey; 2 Department of Medical Statistics and Informatics, Medical School, Bezmialem Vakif University, 34093 Fatih- Istanbul, Turkey

**Keywords:** Quality of life, oral health, reliability and validity, child, preschool.

## Abstract

**Background:**

In Turkey, formal pre-primary education for children 5- 6 years old provides the ideal setting for school-based oral health promotion programs and oral health care services. To develop effective oral health promotion programs, there is a need to assess this target group's subjective oral health needs as well as clinical needs. The Early Childhood Oral Health Impact Scale (ECOHIS) is a well-known instrument for assessing oral health quality of life in children aged 0-5 years old and their families. This study aimed to adapt the ECOHIS for children 5-6 years old in a Turkish-speaking community and to undertake a preliminary investigation of its psychometric properties.

**Methods:**

The Turkish version of the ECOHIS was obtained with forward/backward translations, expert panels and pre-testing and it was tested in a convenience sample of 121 parents of 5- 6 year-old children attending nursery classes of three public schools. Data were collected through clinical examinations and self-completed questionnaires. The main analyses were carried out on the imputed data set. The validity of content, face, construct, discriminant and convergent and as well as the reliability of internal and test-retest of the ECOHIS were evaluated. Sensitivity analysis was performed to examine the effect of the complete case analysis for managing "Don't know" responses on the validity and reliability of the ECOHIS.

**Results:**

The analysis of the imputed data set showed that Cronbach's alphas for the child and family sections were 0.92 and 0.84 respectively, and for the whole scale was 0.93. The intraclass correlation coefficient for test-retest was 0.86. The scale scores on the child and parent sections indicating worse quality of life were significantly associated with poor parental ratings of their child's oral health, high caries experience, higher gingival index scores and problem-orientated dental attendance, supporting its construct, convergent and discriminant validity. Sensitivity analysis showed that the mean imputation method and the complete case analysis did not have differing effects on the validity and reliability of the ECOHIS.

**Conclusions:**

This study provided preliminary evidence concerning validity and reliability of the Turkish version of the scale among 5-6-year-old children. Future studies should be conducted on the ECOHIS to evaluate fully its psychometric properties in both community- based and clinically-based studies among parents of children younger than five. This study provides initial evidence that the ECOHIS aimed at children aged 0-5 years may be a useful tool for assessing the oral health quality of life in 6 year - old preschool children.

## Background

Dental disease, treatment experience and oral health problems can negatively affect the oral health related quality of life of preschool children and their parents. Preschool education constitutes the first step of the Turkish education system and covers the education of the children aged 36-72 and it is elective. According to the 2010 statistics of Ministry of National Education, the early education schooling rate increases with reference to age and schooling rate for 60-72 month-olds is almost 15 times greater than the schooling rate for 36-48 month-olds. Turkey formed its ninth development plan strategy covering 2007-2013 in order to match European Union countries in preschool education. Within the framework of this strategy, a pilot project was initiated in 32 provinces to enroll all 5-year-old children in pre-school education in the 2009-2010 school year [1]. Although the preschool environment, which is an important avenue for reaching and educating Turkish young children, provides the ideal setting for school-based oral health promotion programs and oral health care services, there are neither nationwide oral health promotion nor preventive programs to improve the preschool children's oral health [2,3]. The results of nationwide oral health surveys [4,5] have shown tooth decay to be a serious public health problem for 5-6 year-old children in Turkey. The caries prevalence and caries experience (dmft) in 5-year-olds in 2004 [4] were 70% and 3.7, and in 6-year-olds in 1988 [5] were 84% and 4.4, respectively. At age 5 years, restorative treatment needs was 69% and the most frequent need was one (36%) or multiple surface fillings (38%). In terms of the oral health behaviours of children aged 5 years, it is well known that the utilization of oral health services provided by private and public sector is low to medium and irregular. The oral health care visits are usually problem-oriented and seeking relief from pain/toothache is the main reason given for visiting the dentist [2]. When the position of oral health services in Turkey's Health Care System is analysed, it is clear that resources are primarily allocated to curative care without an underlying oral health policy. Access to oral health services covered by the national health insurance system is limited by factors such as increasing demand for treatment and long waiting lists [3].

In Turkey, most studies have focused on the risk factors for early childhood caries and its behavioral, clinical and microbiological determinants [6-9]. No studies have been reported in the literature concerning the impact of dental caries on oral health related quality life (OHRQOL) in preschool children, although a high prevalence of dental caries in childhood has been described in the literature [4,5]. Clinical paramaters have been used to describe the oral health status and treatment needs among 5-6 year-old children in national oral health surveys of Turkey [4,5]. It is known that traditional methods to measure oral health are based on clinical parameters, which only evaluate the physical conditions based on judgments established by professionals - normative assessment - minimizing the psychosocial consequences of the oral conditions [10]. Thus, in assessing oral health status, there is a need to consider subjective oral health status indicators to measure the functional and psychosocial outcomes of oral disorders [11]. In dental public health, these measurement are useful tools for developing effective oral health interventions and oral health services because they allow determination of population needs, suggest priority of care, and permit evaluation of adopted treatment strategies [12,13]. In order to evaluate the impact of oral health problems and treatments on OHRQOL of children in the 5-6-yr age group (the internationally accepted comparative age group for children), there is a need for a standard instrument which evaluates children's OHRQOL. To date, two instruments have been proposed for this purpose in preschool aged children: the Michigan Oral Health-related Quality of Life Scale [14] and the Early Childhood Oral Health Impact Scale (ECOHIS) [15]. Evidences indicates that children younger than 8 years of age probably cannot recall details of events important to their health more than 24 hours previously [16] and that the child's oral health problems affect not only overall health, but also family welfare, because it results in lost workdays and time and expenditures associated with dental treatment [17]. Therefore, assessing of parents' perceptions about how oral health problems, including symptoms, diseases and its treatment influence their children's oral health and their life, is an important part of measuring young children's OHRQOL [12].

The aim of this study was to develop a Turkish version of the ECOHIS, which is a parent -assessed OHRQOL measure developed to measure the impact of dental caries on children or their families and to evaluate its validity and reliability among 5-6-year-old children.

## Methods

The study was performed in two stages. In the first stage, the scale was translated into Turkish and adapted to Turkish culture. In the second stage, it was tested among the parents of preschool children to assess the stability, internal consistency, discriminant and convergent validity of the Turkish version of the ECOHIS.

The ECOHIS has been developed and validated to assess oral health-related negative impacts in 3-5-year-old children and their families, first in English in the USA [15] and then in French [18], Chinese [19], Farsi [20], and Brazilian [21].

It relies on parental ratings of 13 items grouped in two main parts: part one is the child impact section and part two is the family impact section. In the child impact section, there are four domains: child symptoms (1 item), child functions (4 items), child psychology (2 items), and child self-image and social interaction (2 items). In the family impact section, there are two domains: parental distress (2 items) and family function (2 items). Response categories for each question are rated on a 5-point Likert scale to record how often an event has occurred during the life of the child: 0 = never; 1 = hardly ever; 2 = occasionally; 3 = often; 4 = very often; 5 = don't know. ECOHIS scores were calculated as a simple sum of the response codes for the child and family sections separately, after recoding all "Don't know" (DK) responses to missing. Item scores are simply added to create a total scale score. This system creates a scale score range of 0-52, with higher scores indicating greater impacts and/or more problems. The score for the child and family sections have a possible range from 0 to 36 and from 0 to 16, respectively.

### Turkish adaptation process of the ECOHIS

The ECOHIS was originally developed in English and validated in a sample of 295 parents of 5-year-old children in North Carolina [15]. Therefore, in order to measure the oral health-related negative impacts on preschool children in Turkey, this instrument should be subjected to translation and adaptation to be suited to Turkish use [22]. Based on standard recommendations, the process of cross-cultural adaptation involves several steps: translation from English to Turkish; an initial meeting of the expert panel to produce the first Turkish version; pilot-testing in a convenience sample of 37 parents; a second meeting of the expert panel to produce a new consensus version; back-translation to English; re-evaluation by the expert panel members and by one of the developers of the original scale. The ECOHIS was translated from English to Turkish by two native Turkish-speaking translators with experience in health questionnaire translation. In the first meeting, the expert panel consisted of researchers, one pediatric dentist and one pediatrician who examined the two versions of the scale in order to determine a semi-final translation for testing. This was then reviewed to ensure that the final-translation was fully comprehensible and to verify the cross-cultural equivalence of the source and final version. In addition, the face and content validity of the scale were examined by the expert panel in order to assess the clarity of the item wording. This version was then pilot-tested on a convenience sample of 37 parents of 5-6-year-old children to guarantee sensitivity to local culture and selection of the appropriate wording. In a second meeting, modifications were made according to the comments made by parents and expert panel members in order to clarify the content of the questionnaire. The Turkish consensus version of the scale was obtained and it was then back-translated to English by two independent native English-speaking professional translators. The scale was then re-evaluated for adequacy by the members of the expert panel. The cross-cultural translation and adaptation process ended after this consensus version was sent to the author (Pahel, BT), the original developer of the ECOHIS, for comparison and approval.

### Psychometric testing of the scale

According to quality criteria for measurement properties of health status questionnaires proposed by Terwee et al. [23], at least 50 subjects are necessary for an appropriate analysis of construct validity, reproducibility, responsiveness, and ceiling/floor effects and a minimum of 100 subjects are required to perform internal consistency analysis. The sample size of internal consistency for the Cronbach's alpha was calculated by using Bonnett's Formula [24]: n = {2k (k-1)} (z _α/2 _+z_β_)^2^/In {(1-p_k_)/(1- ρ˜_k)_}^2 ^+ 2. In this formula, k is the number of items, p_k _is the required level for the Cronbach's alpha, and ρ˜_k _is a planning value for the Cronbach's alpha based on prior research, z _α/2 _and z_β _are points on the standard normal distribution exceeded with probability α/2 and β, respectively. We expect the ECOHIS to have a Cronbach's alpha of 0.80 in this study [18], and the required level for the Cronbach's alpha is 0.70. For testing H0: p_k _= 0.70 against a two-sided alternative at α = .05 with power of 0.80 where *k *= 13 and ρ˜_k _= 0. 80, a sample size of 108 subjects would be required. In order to allow a 10% missing data rate due to DK responses [18,21], at least 119 subjects should be invited.

To test the psychometric properties of the Turkish version of the ECOHIS, data were collected from a convenience sample of 121 caregivers and their 5-6 year-old children attending nursery classes of three public schools in Fatih Province of Istanbul City during the 2009-2010 school year. This study was incorporated within the ongoing school oral health promotion program performed by the Dental Public Health Department of Istanbul University. The study protocol was approved by the Turkish Ministry of Education and therefore required no additional Internal Review Board for human experiments ethical committee approval. Verbal consent from the parents of the child was obtained before study participants' examanation. The clinical examinations were carried out by the principal researcher, who assessed caries and gingival health. Caries experience in the primary dentition (dmft) was recorded according to the WHO criteria for visual assessment of dental caries in classrooms [25]. Gingival inflammation was evaluated in all non-exfoliating primary teeth after gentle probing, according to the gingival index by Löe and Silness [26]. In this index, a score of 0 denotes normal gingiva, 1 represents no bleeding but mild inflammation present, 2 represents moderate inflammation and bleeding on probing/pressure, and 3 denotes severe inflammation and spontaneous bleeding.

Face and content validity of the questionnaire were examined by the expert panel in order to assess the clarity of the wording of the items prior to the main study. Reliability was assessed in two ways: internal consistency reliability and test-retest reliability [27]. Internal consistency was evaluated using Cronbach's alpha, alpha if item deleted, and item-total correlation coefficients with Pearson correlation coefficients. Test-retest reliability was assessed using the intraclass correlation coefficient (ICC) calculated by two-way analysis of variance [28] using data from respondents who reported no change in their child's oral health status during the 3-week interval between initial and follow-up assessments. For main statistical analysis, ECOHIS scores were calculated as a simple sum of the response codes for the child and family sections separately, after recoding all DK responses to missing. For those with up to two missing responses on the child section or one missing on the family section, a score for the missing items was imputed as an average of the remaining items for that section, as suggested by Pahel et al [15].

Convergent validity was evaluated based on Spearman's rank order correlations between the ECOHIS scores and the rating of the global oral health rating question, and between the child and family sections of the ECOHIS. Interpretation of correlation coefficients was as follows: r ≤ 0.49, weak relationship; 0.50 ≤ r ≤ 0.74, moderate relationship; and r ≥ 0.75, strong relationship [29]. The oral health rating question asked, "In general, how would you rate the oral health of your child?" The response options for this question were: 1. = Excellent, 2. = Very Good, 3. = Good, 4. = Fair, and 5. = Poor. The underlying hypothesis was that a parent who reported high level of impacts in the scale would be more likely to rate the oral health of his or her child fair or poor. We also hypothesized that the child and family sections of the ECOHIS would be significantly correlated because parents' assessment of their child's oral health is likely to be closely related to parental perceptions of the effect of their child's oral health on the family.

Construct validity was examined by correlating ECOHIS scores with dmft and gingival index scores (Spearman's rank correlations). The priori assumption was that dmft and gingival index scores have a moderate- to high correlation with ECOHIS scores. We expected these relationships to hold for both sections of the ECOHIS.

Discriminant validity was evaluated by comparing ECOHIS scores of groups that differ regarding the child's dental caries status (re-categorised into three categories; "none", "1-3" and "≥4" decayed teeth), and dental attendance patterns (re-categorised into three categories; "never attended", "problem-oriented dental attenders " and " attenders for dental check-ups at least once in two years"). The underlying hypothesis was that parents of children with dental caries would report higher ECOHIS scores (indicating worse OHRQOL) than parents of children free of dental caries and, among children who had problem-oriented dental attendance, that OHRQOL would be worse. We expected these relationships to hold for both sections of the ECOHIS.

Missing data due to DK responses are a significant problem in the field of health quality of life research [30-32]. Considering the management of DK response option, Jokovic et al. [31,32] proposes the following: 1- complete case analysis (excluding subjects with DK responses); 2- use adjusted scores; or 3- drop items from the questionnaire that have high proportion of DK responses. We performed sensitivity analyses to examine the effects of the complete case analysis for managing DK responses on the validity and reliability of the ECOHIS. In our study (n = 121), only 6 subjects had one or two DK responses in the child section. In the main analyses, we used the adjusted score which represents the mean item score of the remaining items for that section as proposed by Pahel et al [15]. We did not choose to drop the items with DK responses, because this method usually used to develop the short form questionnaires [30]. The complete case analysis is the most simple and commonly used method for dealing with DK responses in quality of life research, particularly if the number of deleted incomplete cases is relatively small or if the deleted cases are very similar to the complete cases. However, this method leads to a loss of valuable information and compromises the statistical power of studies with small samples, and also introduces the possibility of bias because of differences between deleted and complete subjects [30-32]. For sensitivity analysis, the new dataset (n = 115) was derived from original data set by using the complete case analysis in which only questionnaires without DK responses were retained for the analysis, and scores were calculated by summing the response codes to the questionnaire items. It is known that DK response option in pediatric health outcome research is associated with parent's socio-demographic characteristics and child's oral health status [30,31]. Thus, socio-demographic characteristics and clinical status of participants who used DK were compared with those who did not using Mann - Whitney U test and Fisher exact test to detect possible bias arising from differences between two groups.

The differences in ECOHIS scores between the three groups were assessed using the Kruskal-Wallis test, followed by the Mann-Whitney U-test with the Bonferroni correction for multiple comparisons. To protect against an inflating Type I error, the Bonferroni adjustment technique was applied, so the level of significance for the post hoc test was adjusted from 0.05 to 0.0167 (0.05 divided by 3) for a two-tailed test. All statistical analyses were performed by using SPSS 15.0 software for Windows (SPSS, Inc., Chicago, IL).

## Results

### Turkish adaptation process of the ECOHIS

The Turkish and English back-translation of the ECOHIS are presented in the Appendix. Some difficulties were encountered regarding the translation of the ECOHIS from English language into Turkish language due to colloquial differences between the two languages. To accomplish an accurate cross-cultural adaptation of the scale, some words had to be modified from the original version. Modifications were made according to the comments made by the expert panel and data obtained in the pilot testing. For example, the fourth item 'difficulty pronouncing any words' was translated to 'difficulty saying any words' to facilitate comprehension. The fifth item, 'missed preschool, day-care or school' was adapted as ' How often could your child not go to crèches, kindergarten or pre-school classes' to provide conceptual equivalence of the item rather than a direct verbal equivalence. Preschool education is given in crèches, nursery school and preschool classes in Turkey. Thus, we had to adopt the terms of 'day-care, preschool or school' to 'crèches, nursery school or pre-school classes'. The sixth item, 'trouble sleeping' was adapted as 'How often could your child not sleep because of dental problems or dental treatments?'. In the seventh item, assessing emotional issues, the phrase 'been irritable or frustrated' was not used colloquially in Turkey. This was replaced by the phrase 'been irritable and troubled'. The thirteenth item, **'**financial impact on your family' was adapted as 'How often has your family had financial problems because of your child's dental problems or dental treatments' because this phrase is usual in Turkish colloquial language.

### Psychometric testing

Table 1 shows the results of descriptive analyses of characteristics of the parents and children in the study sample (n = 121). Of the 121 parents, 77.7% (n = 94) were mothers, 48.8% (n = 59) had formal school education of less than or equal to 8 years, and 66.1% (n = 80) were not in employment. The mean monthly family income was TL 1351 (or $ US 918) monthly. The mean age was of children 5.25 ± 0.43 years. A total of 93 children (76.9%) had one or more decayed teeth, 4.1% (n = 5) had filled teeth, and 52.1% (n = 63) had never visited a dentist. The mean dmft score was 3.87 ± 3.96. The mean gingival index score was 0.36 ± 0.59.

**Table 1:** Parent and child characteristics from the study (n = 121)

Parent & child characteristics	
**Parent demographics**	**n (%)**
Relationship to the child	
Mother	94 (77.7)
Guardian/Grandparent/Other	27 (22.3)
**Educational level**	
Primary or lower	59 (48.8)
Secondary	43 (35.5)
Higher	19 (15.7)
**Employment Status**	
Employed full-time or part-time	41 (33.9)
Unemployed	80 (66.1)
**Monthly Family Income^a ^**, Mean (SD)	1350.66 (605.46)
**Child demographics**	
**Gender**	
Male	56 (46.3)
Female	65 (53.7)
**Age (years)**, Mean (SD)	5.25 (0.43)
**Child's caries experience**	
**dmft score**, Mean (SD)	3.87 (3.96)
**Gingival index score**, Mean (SD)	0.36 (0.59)
**Decayed teeth**	
None	28 (23.1)
1-3	42 (34.7)
≥ 4	51 (42.1)
**Child's dental attendance patterns**	
Never	63 (52.1)
Problem based	22 (18.2)
Regular dental check-ups	36 (29.8)

^a ^Monthly Family Income measured in TRY (Turkish Lira, 1 TRY ≈ 0.516 EUR)

The responses to the ECOHIS items are presented in Table 2. For the child impact section of the ECOHIS, 'irritation or frustration' was the most frequently reported item by the parents (46.3). The items related to 'eating (43.8%)', 'sleeping (43%)', 'pain (40.5%)', 'pronouncing (39.7%)', 'drinking (38.9%)', and 'absence (38.8%)' were also reported often in the child impact section of the scale. Items related to 'feeling upset or guilty', 'financial impact to the family' and 'taking time off from work' were reported frequently in the family impact section of the ECOHIS. However, the distribution of responses to each question was skewed because most participants responded "never". Only 4.95% of participants answered DK to one or two of the questions on the child section. Parents responded DK to questions regarding pain and drinking on the child impact section. DK responses were recoded to missing and missing values for the child impact section were imputed with the mean values of the remaining items for this subscale according to the criterion described the original scale development study [15]. The maximum number of impacts reported was 24 on the child impact section and 12 on the family impact section.

**Table 2:** Distibutions of the ECOHIS responses (n = 121)

Impacts	Never or hardly ever n (%)	Occasionally, often, or very often n (%)	Don't know n (%)
Pain	69 (57)	49 (40.5)	3 (2.5)
Drinking	68 (56.2)	47 (38.9)	6 (4.9)
Eating	68 (56.2)	53 (43.8)	0
Pronouncing	73 (60.3)	48 (39.7)	0
Absence	74 (61.2)	47 (38.8)	0
Sleeping	69 (57)	52 (43)	0
Frustrated	65 (53.7)	56 (46.3)	0
Smiling	102 (84.3)	19 (15.7)	0
Talking	97(80.2)	24 (19.8)	0
Upset	85 (70.2)	36 (29.8)	0
Guilty	95 (78.5)	26 (21.5)	0
Work	106 (87.6)	15 (12.4)	0
Financial	97(80.2)	24 (19.8)	0

Table 3 provides a summary of the descriptive statistics: range, floor effect (proportion with score of 0), mean and standard deviation values. No impacts (floor effects, i.e., the lowest possible score of 0) were reported by 9.6% and 34.7% of parents on the child and family sections, respectively. Floor effects were particularly evident for the 'self image and social interaction (43.8%)', 'child symptoms (27.3%)', and 'child psychology (21.5%)' in the child section, and with respect to family function (52.9%) and parental distress (38%) in the family section. No ceiling effects were observed for either of the two sections (i.e., scores of 36 and 16 on the child and family impact sections, respectively). In examining the internal consistency of the Turkish ECOHIS, we found Cronbach's alpha values of 0.92 and 0.84 for the child impact and family impact sections respectively, and 0.93 for the instrument as a whole. Cronbach's alpha coefficients did not increase by deleting any item. The item-total correlation coefficients ranged from 0.50 to 0.81. The lowest coefficients were related to 'pronouncing (0.50)' and 'work (0.53)', and the highest value belonged to 'sleeping (0.81)'.

**Table 3:** Descriptive distributions of the ECOHIS for different domains (n = 121)

Impacts	Number of items	Possible range	Range	Floor effect (% score 0)	Mean (SD)
Child impact section	9	0-36	0-24	8.3	10.16 (7.12)
Child symptoms	1	0-4	0-4	27.3	1.38 (1.16)
Child function	4	0-16	0-13	10.7	4.90 (3.43)
Child psychology	2	0-8	0-6	21.5	2.48 (1.83)
Self image and social interaction	2	0-8	0-6	43.8	1.38 (1.59)
Family impact section	4	0-16	0-12	34.7	2.88 (3.17)
Parental distress	2	0-8	0-7	38	1.69 (1.86)
Family function	2	0-8	0-6	52.9	1.19 (1.64)
Total score	13	0-52	0-36	8.3	13.04 (9.61)

SD, standard deviation

The test-retest reliability of the Turkish ECOHIS was examined through a sub-sample of the study sample completing the scale a second time three weeks after the first completion. No change in health status was reported by 23 out of 30 (76.6%) participants who returned the instrument with complete responses. ICC values were 0.86 for the whole scale, 0.83 for the child impact section and 0.90 for the family impact section.

Both hypotheses regarding convergent validity were confirmed. We investigated the Spearman correlation coefficient for the global oral health rating and total ECOHIS score and found a moderate correlation (r = 0.68; P < 0.01). The correlations for the global ratings with the child and family impact sections of the ECOHIS were r = 0.70 (P < 0.01) and r = 0.52 (P < 0.01) respectively (Table 4). The correlation between the child and family impact sections was statistically significant (r = 0.68, P < 0.01).

**Table 4:** Findings for convergent and construct validity of the ECOHIS score using two methods to manage "Don't know" responses

	Mean imputation method (n = 121)			Complete case analysis (n = 115)		
	Child Section	Family section	Total score	Child Section	Family section	Total score
	**r**	**r**	**r**	**r**	**r**	**r**
Global oral health rating	0.70**	0.52**	0.68**	0.71**	0.53**	0.69**
dmft	0.71**	0.72**	0.77**	0.71**	0.73**	0.78**
GI	0.65**	0.68**	0.71**	0.68**	0.70**	0.73**

r, Spearman's correlation coefficient

** Statistically significant at P < 0.01

As shown in Table 4 the ECOHIS scores were significantly correlated with dmft (r = 0.77, P < 0.01) and gingival index scores (r = 0.71, P < 0.01). These findings provide support for construct validity of the ECOHIS.

Hypotheses concerning discriminant validity were confirmed - that is, there were significant differences in child and family sections scores among the groups classified according to the dental attendance patterns and the number of decayed teeth in children (Table 5). The results of the Mann Whitney U test with Bonferroni correction showed that overall, caries-free children and those with 1-3 decayed teeth had lower scores on the child and family sections of the ECOHIS than those who had ≥ 4 decayed teeth (P < 0.0167). Further, we found that problem-oriented attenders had higher scores on the child and family sections of the ECOHIS than those with regular dental attendance patterns and without a dental visit (P < 0.0167). Sensitivity analyses showed similar directions of results obtained from the imputed data. There were no statistically significant differences between participants with and without DK responses in education level, employment status and monthly family income as well as in child's primary caregiver, gender, and age. The differences among groups for child's dmft and gingival indices were not statistically significant (results not shown). Cronbach's alpha coefficient of the ECOHIS and its child and family sections were 0.93, 0.92, and 0.85 respectively. Cronbach's alpha coefficients did not increase by deleting any item. The item-total correlation coefficients were ranged from 0.51 to 0.81. ICC values for test-retest were 0.86 for the whole scale, 0.83 for the child impact section and 0.90 for the family impact section. The complete case analysis and the mean imputation method did not have differing effects on Cronbach's alpha values and ICC values for the whole scale and for both child and family impact sections.

**Table 5:** Findings for discriminant validity of the ECOHIS score using two methods to manage "Don't know" responses

		Mean imputation method (n = 121)			Complete case analysis (n = 115)	
Variables		Child Section	Family section		Child Section	Family section
**Number of decayed teeth^a^**	**n**	**Mean (SD)**	**Mean (SD)**	**n**	**Mean (SD)**	**Mean (SD)**
None (A)	28	5.57 (4.28)^C^*	0.67 (1.02)^C^*	27	5.37 (4.23)^C^*	0.63 (1.00)^C^*
1-3 (B)	42	5.33 (4.30)^C^*	1.09 (1.58)^C^*	38	5.02 (4.30)^C^*	1.00 (1.62)^C^*
≥ 4 (C)	51	16.66 (4.71)^A, B^*	5.56 (2.95)^A, B^*	50	16.68 (4.76)^A, B^*	5.64 (2.93)^A, B^*
P - value		< 0.001	< 0.001		< 0.001	< 0.001
**Child's dental attendance pattern**^a^		**Mean (SD)**	**Mean (SD)**		**Mean (SD)**	**Mean (SD)**
Never (A)	63	8.33 (6.87)^B^*	2.14 (2.07)^B^*	60	8.23 (6.96)^B^*	2.16 (2.11)^B^*
Problem based (B)	22	14.45 (6.26)^A, C^*	5.27 (3.52)^A, C^*	21	14.52 (6.41)^A^*	5.42 (3.52)^A, C^*
Regular dental check - ups (C)	36	10.75 (6.99)^B^*	2.72 (3.85)^B^*	34	10.91 (7.13)	2.73 (3.96)^B^*
P-value		0.002	0.001		0.002	0.001

SD, standard deviation

^a ^Statistical evaluation by the Kruskal-Wallis test

*Significant differences among groups according to Mann-Whitney U-test with Bonferroni correction (P < 0.0167)

The analyses of convergent, discriminant and contruct validity using complete data set scores confirmed all hypotheses. The correlation between the scores obtained on the child and family impact sections was statistically significant (r = 0.69, P < 0.01). As shown in Table 4, correlation coefficients between the global oral health rating and the ECOHIS total score, child section and family section were 0.69, 0.71, and 0.53, respectively. The ECOHIS scores were significantly correlated with dmft (r = 0.78, P < 0.01) and gingival index scores (r = 0.73, P < 0.01). We found similar significant differences in child and family sections scores among the groups classified according to the dental attendance patterns and the number of decayed teeth in children, supporting discriminant validity of the ECOHIS (Table 5).

## Discussion

To develop effective oral health promotion interventions and oral health care services for Turkish preschool aged children, there is a need for the standard and validated measurement to assess children's oral-health-related quality of life [33].

The ECOHIS has been previously validated and used in different countries [18-21]. As with many such instruments, this scale was developed in English and requires translation and validation in other languages if it is to be used in these languages. In the present study, the original English-language ECOHIS was translated into Turkish, following the recommendations of Guillemin et al. [22] and resulted in a back-translated version that was very similar to the original although word modifications were made to take into account of cultural differences. The Turkish version of the ECOHIS exhibited acceptable validity and reliability.

In relation to internal consistency, the item-total correlation values were higher than the recommended 0.20 and alpha decreased when any item was deleted. Cronbach's alpha of this study was satisfactory (0.93, 0.92, and 0.84 for the ECOHIS, child section, and family section respectively) as it follows the standards for acceptable reliability of Cronbach's alpha [27]. Cronbach's alpha values were close to those of the original English questionnaire [15] and Farsi version of ECOHIS [20], and higher than the French [18], Chinese [19], and Brazilian [21] versions of ECOHIS. In the test-retest reliability, the ICC for the total scale was 0.86 and ranged from 0.83 to 0.90 for the sections, indicating good reproducibility [28] but less than that reported in the French and Brazilian validation studies [18,21]. It was higher than the values of Pahel et al. in USA [15], those of Jabarifar et al. in Iran [20], and those of Lee et al. in China [19].

The majority of parents (91.7%) reported that their child experienced at least one oral health impact, mostly child functional and psychological impairments. An impact on the family as a result of the child's oral health was reported by 65.3% of parents.

In contrast to the findings by Pahel et al. [15] and Li et al. [18], 8.3% of parents in this study reported no impact of oral health problems on their children's quality of life. In this respect, it is important to note that the results obtained using the Turkish ECOHIS are similar to those obtained using the Chinese version, which also had a low floor effect. This is probably indicative of the subjects having high levels of problems, although our study population was a convenience sample comprised of parents whose children attend the oral health promotion programs. This may be explained by the fact that caries experience among 5-6-year-olds children is high and only 2.1% of 5-6 year olds have filled teeth in Turkey [4,5]. No ceiling effect was detected, consistent with other validation studies [15,18]. Analyzing the distribution of items in this study, the most frequently reported items on the two sections of the scale were practically the same as those reported in previous validation studies of ECOHIS [15,18,19,21]. On the child impact section, the most prevalent items were related to 'irritation or frustration', 'eating', 'sleeping', and 'pain'. On the family impact section, the most prevalent item was 'feeling upset or guilty'.

As done in previous studies [15,18,21], the number and distribution of DK responses were taken into account in the main analyses, because DK response option is important, particularly during the validation phase of instrument development and use, so as to have an indication of the pertinence and comprehensibility of the items [30]. In addition, this response option is essential in studies in which participants report their perceptions of the health or quality of life of another individual, as it reflects a particular characteristic of the phenomenon under evaluation. In addition, parents' knowledge of their children's health-related quality of life could be explored by examining the frequency and distribution of DK responses to the questionnaire items [30,31]. In our study, 4.95% parents answered DK to one or two of the questions only on the family section, which is lower than that reported from studies carried out in the USA [15], France [18] and Brazil [21]. This study showed that Turkish parents' knowledge concerning their children's disease-related experiences such as 'pain' and 'drinking' is limited consistent with previous studies [15,18,21].

Evidence for discriminant validity of the ECOHIS is provided by the finding of higher ECOHIS (indicating worse OHRQOL) scores on both sections among those with more than 4 decayed teeth compared with those who were caries free or had 1-3 decayed teeth. This finding is in agreement with a Brazilian study [21], which reported that children with dental caries experience, those with more severe dental disease obtained higher ECOHIS scores than those without dental caries and those with less severe dental disease. Consistent with our findings, Pahel et al. [15] also found similar associations only in the child section and Lee et al. [19] found a significant difference between children with caries and those without caries in both sections. Consistent with the findings of Lee et al. [19], we found that problem-orientated attenders had higher scores on the both sections of the ECOHIS than those with regular dental attendance patterns or who did not visit a dentist.

Regarding convergent validity, the Turkish version of the ECOHIS scale showed a moderate correlation with the global rating of oral health. This finding was consistent with previous studies [15,18,20,21] reporting that parents who thought their children had worse oral health were more likely to give their children higher ECOHIS scores. Additionally, this finding supports suggestions that parents can provide valid reports for their preschool children's OHRQOL when these conditions are observable [14,15]. Consistent with previous studies, we found a strong correlation between child and family items of the scale, indicating that the ECOHIS is strongly associated with the underlying construct of OHRQOL [15,18-21]. Moderate positive correlations were observed among caries experience, gingival index scores and ECOHIS scores. These findings support the construct validity of the measure. It should be noted that researchers investigated the association only among the ECOHIS scores, dmft [15,19,21] and discolored upper anterior teeth [21] in previous validation studies, when testing the construct validity of the ECOHIS. In this study, gingival health was measured using the gingival index score as clinical indicator, because gingivitis is an inflammatory process that begins about the age of 5 years [34,35].

There are three suggested methods to handle missing data due to DK responses. In the main analyses, the mean imputation method was applied because only 6 subjects had ≤ 2 DK responses in the child section. Pahel et al. [15]. suggest that DK-responses are replaced with the personal mean on that particular section for subjects with up to two missing responses on the child section or one missing on the family section and use of this criterion may increase possibility to include more participants in the analysis. We did not choose to drop the items with DK responses, because DK response reflects an essential characteristic of the phenomenon being measured rather than a limitation in the questionnaire. In order to assess the impact of missing data on our findings, we examined the results when performing the analyses using mean imputation method, and when using the complete case analysis. Complete-case analysis was preferred because the number of deleted incomplete cases was relatively small and the deleted cases were very similar to the complete cases in terms of socio-demographic and clinical factors. Sensitivity analysis showed that the mean imputation method and the complete case analysis did not have differing effects on the validity and reliability of the ECOHIS. The results support previous evidence that excluding subjects or using adjusted scores did not affect the validity analyses [30,31].

Psychometric testing of the scale demonstrated good convergent, construct and discriminant validity as well as internal consistency and test-retest reliability in the imputed data set, as well as the complete data set. There were some limitations to the study. One of the limitations of the study is the use of the ECOHIS in 5-6-year old preschool children because this measure was developed and validated for use in 0-5 years- old- children [15,18-21]. This study provided preliminary support for psychometric properties of the Turkish version of ECOHIS in consecutive samples consist of parents of 5-6-year-olds. Therefore, our results provide evidence for its performance in this population only. Future studies should be conducted on the ECOHIS to evaluate fully its psychometric properties in both community- based and clinically-based studies among parents of children younger than five. Its sensitivity to change should also be established, so that it can be considered for clinical trials to assess the effect of dental disease and its treatment on quality of life [36]. It should be noted that the Turkish version of the ECOHIS was validated by using classical test theory used in previous validation studies [15,18-21]. Recent study used Rash analysis reported that the Chinese version of ECOHIS has a range of difficulty levels across the items and performance of item consistency and these results reinforce the need to analyse the existing translations of ECOHIS [37]. Future study using Rash analysis may provide additional information to the classical test theory and allow for the examination both of individual item's difficulty level and discriminatory ability [37,38].

## Conclusions

Based on this preliminary study's results, the following conclusions can be made:

The Turkish version of ECOHIS is a reliable and valid instrument for assessing the OHRQOL in 5- 6- year old pre-school children of the studied community.

The use of this scale could help clinicians, researchers and policymaker to describe the effects of dental disease and treatment experience on young children and their families and to plan effective oral health promotion interventions and oral health care services.

This scale could provide the opportunity to compare similarities and differences in oral health impacts among young children in different countries.

The results of sensitivity analysis support previous evidence that excluding subjects or using adjusted scores did not affect the validity and reliability analyses of this scale.

This study provides initial evidence that the ECOHIS aimed at children aged 0-5 years may be a useful tool for assessing the oral health quality of life in 6 year - old preschool children.

## Abbreviations

ECOHIS: The Early Childhood Oral Health Impact Scale; dmf-t: The number of decayed, missing and filled deciduous teeth; OHRQOL: Oral health related quality life; USA: The United States of America; ICC: The intraclass correlation coefficient; DK: Don't know.

## Competing interests

The authors declare that they have no competing interests.

## Authors' contributions

KP conceptualized and designed the study, acquired, interpreted the data, drafted the manuscript, and wrote the paper. GB contributed to the data collection and the study management. ÖU contributed to the data analysis and interpretation. All authors read and approved the final manuscript.

## Appendix. Turkish and English back- translation of the ECOHIS

### Erken Çocukluk Çağı Ağız Sağlığı Etki Ölçeği*

Dişler, ağız ve çenelerle ilgili problemler ve onların tedavisi çocukların ve ailelerinin günlük yaşamlarını ve iyilik hallerini etkileyebilir. Lütfen, aşağıdaki soruların her biri için, çocuğunuzun ve sizin deneyimlerinizi en iyi tanımlayan yanıt seçeneğinin yanındaki kutucuğu "X" ile işaretleyiniz. Her soruyu cevaplarken, çocuğun doğumdan günümüze kadar olan tüm yaşamını dikkate alın.

Çocuğunuzun dişlerinde, ağzında veya çenelerinde ne sıklıkta ağrısı oldu?

Çocuğunuz diş problemleri ve tedavileri nedeniyle, ne sıklıkta sıcak ve soğuk içecekleri içmede zorluk yaşadı?

Çocuğunuz diş problemleri ve tedavileri nedeniyle, ne sıklıkta bazı yiyecekleri yemede zorluk yaşadı?

Çocuğunuz diş problemleri ve tedavileri nedeniyle, ne sıklıkta herhangi bir kelimeyi söylemede zorluk yaşadı?

Çocuğunuz diş problemleri ve tedavileri nedeniyle, ne sıklıkta kreşe, anaokuluna veya anasınıfına gidemedi?

Çocuğunuz diş problemleri ve tedavileri nedeniyle, ne sıklıkta uyuyamadı?

Çocuğunuz diş problemleri ve tedavileri nedeniyle, ne sıklıkta sinirli ve husursuz oldu?

Çocuğunuz diş problemleri ve tedavileri nedeniyle, ne sıklıkta gülümsemekten ve kahkaha atmaktan çekindi?

Çocuğunuz diş problemleri ve tedavileri nedeniyle, ne sıklıkta konuşmaktan çekindi?

Çocuğunuzun diş problemleri ve tedavileri nedeniyle, siz veya diğer aile bireyleri ne sıklıkta rahatsız oldu?

Çocuğunuzun diş problemleri ve tedavileri nedeniyle, siz veya diğer aile bireyleri ne sıklıkta kendini suçlu hissetti?

Çocuğunuzun diş problemleri ve tedavileri nedeniyle, siz veya diğer aile bireyleri ne sıklıkta işinden izin aldı?

Çocuğunuzun diş problemleri ve tedavileri nedeniyle, siz veya diğer aile bireylerinin ne sıklıkta maddi problemleri oldu?

### The Early Childhood Oral Health Impact Scale (ECOHIS)*

Problems with the teeth, mouth or jaws and their treatment can affect the well-being and daily life of children and their families. For each of the following questions, please mark an "X" in the box next to the response that best describes your child's experiences or your own. Consider the child's whole life from birth until now when answering each question.

How often has your child had pain in the teeth, mouth or jaws?

How often has your child had difficulty drinking hot or cold beverages because of dental problems or dental treatments?

How often has your child had difficulty eating some foods because of dental problems or dental treatments?

How often has your child had difficulty saying any words because of dental problems or dental treatments?

How often could your child not go to crèches, kindergarten or pre-school classes because of dental problems or dental treatments?

How often could your child not sleep because of dental problems or dental treatments?

How often has your child been irritable or troubled because of dental problems or dental treatments?

How often has your child avoided smiling or laughing because of dental problems or dental treatments?

How often has your child avoided talking because of dental problems or dental treatments?

How often have you or another family member been upset because of your child's dental problems or dental treatments?

How often have you or another family member felt guilty because of your child's dental problems or dental treatments?

How often have you or another family member taken time off from work because of your child's dental problems or dental treatments?

How often has your family had financial problems because of your child's dental problems or dental treatments?

* The choice of alternatives in the self-administered first part of the scale was supposed to be marked with a check mark or an 'x' in a box for each item. In Turkey it is more common to use an "X" than using a check mark, so the "X" was adopted in the Turkish version.
